# Sociocultural factors contributing to waterpipe tobacco smoking among adolescents and young adult women: a qualitative study in Iran

**DOI:** 10.1080/17482631.2020.1857043

**Published:** 2021-01-12

**Authors:** Zeinab Makvandi, Firoozeh Mostafavi, Saeed Bashirian, Fereshteh Zamani-Alavijeh, Roya Kelishadi

**Affiliations:** a Student Research Committee, School of Heath, Isfahan University of Medical Sciences, Isfahan, Iran; b Department of Health Education and Promotion, School of Health, Isfahan University of Medical Sciences, Isfahan, Iran; c Social Determinants of Health Research Center, Hamadan University of Medical sciences, Hamadan, Iran; d Child Growth and Development Research Center, Research Institute for Primordial Prevention of Non-Communicable Disease, Isfahan University of Medical Sciences, Isfahan, Iran

**Keywords:** Waterpipe tobacco smoking, adolescents, young adult, women, qualitative study

## Abstract

**Purpose**: Waterpipe tobacco smoking (WTS) is currently a serious and growing public health threat in the world, especially in adolescents and young women. The aim of the study was to explore sociocultural factors contributing to WTS among adolescents and young adult women in Iran.

**Methods**: This qualitative study was conducted from August 2017 to January 2019 in Isfahan and Hamadan cities, Iran; it included 13–30-year-old females with experience of WTS. For data collection, in-depth semi-structured personal interviews were conducted at participants’ preferred time and place. Concurrent with data collection, data were analysed through conventional content analysis.

**Results**: The study participants described the various sociocultural factors contributing to WTS. These factors were categorized into four following main categories: waterpipe glamorization by its producers and sellers, media advertisement or silence, common sociocultural traditions, and governmental policies and regulations.

**Conclusion**: A wide range of sociocultural factors affects WTS among Iranian adolescents and young adult women. Therefore, interdisciplinary multidimensional strategies are needed for WTS management and prevention among these at-risk groups. Public education, strict supervision of tobacco import, export, and selling, ample employment opportunities for young people and effective leisure time management are essential to reduce WTS.**Abbreviation WTS**: Waterpipe Tobacco Smoking; MPOWER: Monitor tobacco use and prevention policies, Protect people from tobacco smoke, Offer help to quit tobacco use, Warn about the dangers of tobacco, Enforce bans on tobacco advertising, promotion and sponsorship, Raise taxes on tobacco

## Background

Tobacco smoking is a major health challenge accounting for more than seven million deaths throughout the world (WHO, 2015, 2018). Waterpipe tobacco smoking (WTS) is a traditional method for tobacco smoking. Because of its progressively increasing prevalence, particularly among adolescents and young adult people, WTS has turned into a serious global public health threat (Aboaziza & Eissenberg, 2015; Kim et al., 2016). The highest prevalence of WTS among young people is in the Eastern Mediterranean region. Its prevalence in this region ranges from 2.5% in Oman to 37.2% in Lebanon. WTS prevalence in Europe also ranges from 2.2% in Romania to 22.7% in Lithuania. The lowest prevalence of WTS is in the USA, where it ranges from 1.0% to 11.4% (Jawad et al., 2018).

WTS is associated with different negative outcomes including lung and mouth cancers, cardiovascular disease, hyperglycaemia, respiratory disorders, sex hormone disorders, osteoporosis, and low birth weight (Akl et al., 2010; Waziry et al., 2017; Wong et al., 2016). It also predisposes women to cervical cancer, ectopic pregnancy, spontaneous abortion, premature delivery, and other reproductive problems (Caleyachetty et al., 2014; Tarney et al., 2018). Estimates show that tobacco smoking will cause 8.3 worldwide deaths in 2030, with 70% being in developing countries (WHO, 2015, 2018).

Despite the serious health outcomes of WTS, its popularity has significantly boosted among adolescent and young adult women (Daou et al., 2018; Maziak et al., 2017). Studies reported that its prevalence among young women is 6.3% in the USA (Salloum et al., 2015) and 41% in Pakistan (Khan et al., 2015). WTS is also a significant public health challenge in Iran and is the most common method for tobacco smoking by adolescent and young adult women (Baheiraei et al., 2015b; Mojahed & Navidian, 2018; Ziaei et al., 2016). Studies in Iran reported that WTS prevalence ranged from 7.1% (Mohammad-Alizadeh-Charandabi et al., 2015) to 38.9% (Baheiraei et al., 2013) among adolescents and was 11.5% among female university students (Roohafza et al., 2011).

The World Health Organization has emphasized on the identification of WTS gender-specific contributing factors (WHO, 2010); nonetheless, little efforts have been made so far in this area. A handful of studies in this area reported that WTS contributing factors include personal factors such as curiosity, positive attitude towards WTS, and personal pleasure, as well as interpersonal and environmental factors such as lack of emotional family support, WTS by family members, peer pressure, lack of healthy recreational activities, inexpensiveness and easy accessibility of WTS, and lack of public educations against WTS (Akl et al., 2015; Gathuru et al., 2015; Jawad et al., 2015; Mao et al., 2014; Nakkash et al., 2011; Ramji et al., 2015; Roman et al., 2017; Villanti et al., 2015). The results of studies on women’s perceptions of WTS were different from the results of studies on men. For instance, a multinational study in the Eastern Mediterranean region reported that the sexual allure associated with WTS and considering WTS as a symbol of emancipation were the main factors affecting WTS among women (Khalil et al., 2013). Another study in Iran found that considering WTS as a weight-losing strategy contributed to girls’ and women’s resort to its use (Majdzadeh et al., 2002). These findings denote the wide variety of factors that may result in WTS among women. Some studies also indicated that while men use tobacco mostly out of habit or to have positive feelings, women use it to manage their negative emotions or stress and/or to regain calmness (Kin & Lim, 2004; Oh et al., 2010; Salameh et al., 2012). Beliefs and attitudes can also affect WTS (Arshad et al., 2019). WTS-contributing beliefs and attitudes may be transferred to girls and women by their families, friends, or social environment (Baheiraei et al., 2015a). For instance, some cultures have better acceptance on waterpipe smoking in women compared with cigarette smoking (Akl et al., 2015). Nonetheless, there is limited information about the role of sociocultural factors contributing to WTS among women. Therefore, studies in different contexts are needed to provide a deeper understanding of these factors. The present study was conducted to narrow these gaps. The aim of the study was to explore sociocultural factors contributing to WTS among adolescent and young adult women in Iran.

## Methods

### Study design

As part of a larger study, this qualitative study was conducted from August 2017 to January 2019 using conventional content analysis approach. Conventional content analysis is a systematic approach for providing detailed description of phenomena. This approach is appropriate for exploring individuals’ direct experiences of phenomena and is mostly useful for poorly known phenomena (Elo & Kyngäs, 2008). Therefore, in the current study, this approach was used to provide an in-depth understanding about sociocultural factors contributing to WTS among Iranian adolescents and young adult women.

### Participants and data collection

Study participants were adolescent and young adult women who had the experience of WTS and lived in Isfahan and Hamadan, Iran. Inclusion criteria were being aged 13–30 years and agreement to participate in the study and to share WTS-related experiences. However, to identify the diverse aspects of the WTS among adolescents and young adult women and increase the strength and consistency of the data, people who were somehow connected with this the issue are also eligible to participate in the interviews. Other participants included family members, friends, WTS providers, teachers, and smoking control staffs. Participants were selected with maximum variation from different educational levels, employment status, ethnic groups, and geographic areas in Iran. Sampling was done through both purposeful and snowball sampling and was continued up to data saturation. Less than 10% of eligible participants who had been invited to the study refused participation.

Data were collected via in-depth semi-structured interviews opened using questions on participants’ demographic characteristics such as age, educational level, marital status, occupation, age at first WTS, family history of tobacco use, and WTS pattern. Then, interviews were continued using general open-ended questions about WTS such as “May you please explain about your WTS?” “What factors made you to smoke tobacco using waterpipe?” “What factors would make you to continue smoking tobacco using waterpipe?” Based on participants’ responses to such questions, probing questions were asked to collect more in-depth data. Examples of probing questions were, “May you please explain more about this?” and “What do you mean by this?”. Moreover, based on the data extracted from the initial interviews, questions about sociocultural factors contributing to WTS were used in later interviews. Examples of these questions were as follows: “What social factors can affect your WTS?” “What is your opinion about the effects of advertisements for WTS?” Questions used for other participants were different from those used for main participants. For instance, a question for WTS providers and sellers was as follows: “What measures do you take to attract more female customers and tempt them into WTS?” while two questions for smoking control staff were as follows: “What are the guidelines for controlling WTS” and “How well are these guidelines being followed?”Interviews were conducted at participants’ preferred time and place, including recreational facilities, hookah bars, workplaces, and dormitories. The length of interviews ranged from thirty to 65 minutes. Although the social stigma associated with women’s WTS is less severe than the stigma associated with their cigarette smoking, some women may prefer not to openly talk about their WTS (‏ Baheiraei et al., 2015c). Moreover, some participants refused talking about their WTS in focus group discussions due to personal preferences or their husbands’ or parents’ disagreement. Consequently, all interviews were held in-person by a female interviewer (i.e. the first authors) in order to gain the participants’ trust. Forty participants provided consent for recording their interviews singed a digital voice recorder, while two participants refused such consent and hence only their interviews were only documented.

### Data analysis

Concurrent with data collection, data analysis was performed through the three-phase conventional content analysis. In the data preparation phase, the interviews were transcribed word by word and reviewed for several times for data immersion. In the data organization phase, primary codes were inductively identified and grouped into categories according to their conceptual similarities. Categories were labelled based on their contents. Constant comparison of the data and the findings helped revise or combine the categories and develop new categories. This process of data reduction resulted in the identification of the final pattern in the data. Finally, the findings were reported in the data reporting phase (Elo & Kyngäs, 2008).

### Rigour

Transferability was ensured through maximum variation sampling, while credibility and dependability were ensured through prolonged engagement with the data. Member checking was also performed to ensure the congruence between findings and participants’ experiences. Moreover, through peer and external checking, the transcripts of some interviews and their corresponding codes were appraised by several experts in qualitative research (Polit & Beck, 2004).

### Ethical considerations

The Ethics Committee of Isfahan University of Medical Sciences, Isfahan, Iran, approved this study (IR.MUI.REC.1396.3.46). At the beginning of each interview, the aim and the methods of the study were explained to the intended participant and they was ensured of the voluntariness of participation in the study, confidentiality of the study data, and her freedom to unilaterally withdraw from the study. Informed consent was obtained from all participants. Moreover, informed verbal or written consent was obtained from a parent or guardian for participants with less than 16 years of age.

## Results

Overall, 42 persons participated in this study. Thirty-four participants were adolescent and young adult women with WTS experience and with mean (SD) age of 22.97 (4.89) years, on average. Twenty-nine of these women (85%) still smoked waterpipe tobacco at the time of the study, while five of them had ceased WTS. The remaining eight participants were two mothers, one girlfriend, and one boyfriend, two male waterpipe tobacco smokers who worked in hookah bars, one female teacher, and a male smoking control staff. Their mean (SD) age of years was 34 (9.95). Table 1 shows participants’ demographic characteristics.

**Table 1.** Participants’ demographic characteristics

Participants Characteristics		Main (N = 34)* N (%)	Other participants (N = 8)** N (%)
Age (year)	13–18	8 (23.5)	0
	19–24	13 (38.3)	2 (25.0)
	25–30	13 (38.2)	1 (12.5)
	> 30	0	5 (62.5)
Educational level	Below diploma	8 (23.5)	1 (12.5)
	Diploma	4 (11.8)	2 (25.0)
	University	22 (64.7)	5 (62.5)
Marital status	Single	26 (76.5)	2 (25.0)
	Married	6 (17.6)	6 (75.0)
	Divorced/widowed	2 (5.9)	0
Employment status	Employed	12 (35.3)	3 (37.5)
	Unemployed	22 (64.7)	5 (62.5)
WTS pattern	Current user	29 (85.3)	2 (25.0)
	Former user	5 (14.7)	1 (12.5)
	Non-user	0	5 (62.5)
Age at first WTS	5–10	1 (2.9)	0
	11–20	30 (88.3)	1 (12.5)
	21–30	3 (8.8)	2 (25.0)
	Non-user	0	5 (62.5)
Family history of any tobacco use	Yes	20 (58.8)	4 (50.0)
	No	14 (41.2)	4 (50.0)

*Main participants: adolescent and young adult women aged 13–30 years with a positive history of WTS

**Other participants: Individuals who had experiences related to main participants’ experiences. Other participants included family members, friends, WTS providers, teachers, and smoking control staffs.

Sociocultural factors contributing to WTS were categorized into four main categories. These categories were waterpipe glamorization by its producers and sellers, media advertisement or silence, common sociocultural traditions, and governmental policies and regulations. Together with their subcategories, these categories are shown in Table 2 and are explained in the following.

**Table 2.** Sociocultural factors contributing to waterpipe tobacco smoking among adolescent and young adult women

Subcategories	Categories
Innovative and tempting techniques for the continuous glamorization of waterpipe by its producers	Waterpipe glamorization by its producers and sellers
Marketing and customer attraction techniques used by WTS providers	
Media advertisement for WTS	Media advertisement or silence
Mass media silence respecting WTS	
WTS as a norm transgression	Common sociocultural traditions
Social acceptance of WTS by women	
Ineffective enforcement of WTS-related regulations	Governmental policies and regulations
Inadequate supervision of tobacco production, supply, import, and export	

### Waterpipe glamorization by its producers and sellers

This main category consisted of two subcategories, namely innovative and tempting techniques for the continuous glamorization of waterpipe by its producers, and marketing and customer attraction techniques used by WTS providers.

#### Innovative and tempting techniques for the continuous glamorization of waterpipe by its producers

Waterpipe producers continuously attempt to design and produce as attractive as possible waterpipe and waterpipe accessories in order to tempt different individuals into WTS.

Using a single type of waterpipe is boring. I have two waterpipes. One of them is like the Eiffel tower and the other is smaller. I use them intermittently. Every day, they produce new types of waterpipe which I like. (Current smoker, 28year old)

The wide variety of tobaccos and their flavours is also a significant factor contributing to WTS among adolescent and young adult women. Participants considered the different aromas used for tobaccos as a tempting factor for their WTS.

I ceased WTS; but this new tobacco, which has a pleasant aroma, tempted me into its use, particularly with these new waterpipes. (Former smoker, 26 year old)

#### Marketing and customer attraction techniques used by WTS providers

The participating waterpipe tobacco smokers, hookah bar staff, and smoking control staff noted that hookah bars compete with each other for attracting more customers and hence, use different marketing and customer attraction techniques based on their customers’ personal preferences. One of these techniques was to offer a wide variety of tobaccos with different flavours and waterpipes with different shapes.

There are different tobaccos with different fruit flavors. We offer them to our customers in order to satisfy them. (The staff of hookah bars, male, 27 year old)

Some participants referred to the beautiful interior decoration and the pleasant environment of hookah bars as significant factors contributing to WTS by adolescent and young adult women.

The interior decoration of hookah bars is really luxury. They use colorful or wooden decorations and use different colors for decoration. Such decoration tempts me into going there and smoking waterpipe. (Former smoker, 20 year old)

The staff of a luxury hookah bar said,

Here, we have designed rooms specific to women where we have used women’s favorite colored lighting such as pink and red lighting. Moreover, we offer glittery and attractive waterpipes. For example, we have a waterpipe specific to women that has the shape of a butterfly whose wings moves with each puff. Its hose also has glittery and acrylic texture. Girls like these attributes and hence, they come here more often. (The staff of hookah bars, male, 27 year old

The staff of another hookah bar in a beautiful fourteen-story tower said,

Our bar is in the top of the tower and has a good view over almost the whole city. Moreover, we have installed an elevator with a glass cabin to make our bar more interesting. Therefore, many girls come hear each day to take film for their Instagram stories and smoke waterpipe. (The staff of hookah bars, male, 37 year old)

Some participants noted that special discounts on waterpipe, for instance, discount for students, tempt even those people who do not intend to smoke waterpipe into WTS.

We had gone to a traditional restaurant for launch. We never intended to smoke waterpipe. However, they offered discount on waterpipe for students and hence, we tested waterpipe for the first time. (Current smoker, 22 year old)

In addition to waterpipe, hookah bars offer different services to attract customers. Examples of these services are traditional foods, different drinks, confections, nuts, free high-speed internet, and live music. Such holistic approach to service delivery satisfies different needs of customers at a single time and place and hence, causes them to refer to those places again.

Here, we both enjoy live music, use free high-speed internet, have our foods, and smoke a nice waterpipe after food. In addition, they serve us with confections and nuts. We also drink tea with candy in order to prevent blood pressure decline. With all these services at a single place, why should we go to another place? (Current smoker, 16 year old)

As another customer attraction technique, hookah bars also provide their customers with a safe and private place for other recreations such as intimate circles.

Bowers in hookah bars provide you with a private place to be with friends. There, you can hold celebrations without any disturbance. Certainly, we also need to order waterpipe. (Current smoker, 17 year old)

Another customer attraction technique of hookah bars is to provide a place for hidden norm violation. Hookah bars attempt to attract more customers by offering private places for WTS.

I have to come here to have a private place to be with my boyfriend. Here, they offer us waterpipe and we have no option but to use it though my boyfriend and I are not interested in WTS. (Current smoker, 23 year old)

The other technique used by hookah bars is telephone-based waterpipe delivery services locally called “Hallo waterpipe”. Such services make WTS easily accessible.

Hallo waterpipe is a new service which has recently introduced. You order a ready-to-use waterpipe over the phone and they bring it to your address. You can use it for several hours. (Current smoker, 16 year old)

### Media advertisement or silence

The two subcategories of this main category were media advertisement for WTS and mass media silence respecting WTS.

#### Media advertisement for WTS

According to the participants, WTS advertisement, particularly in non-official media such as the Instagram and Telegram, is sometimes effective in tempting young women into WTS.

For the first time, I heard about the “Hallo waterpipe” service in the Instagram. Afterwards, whenever I was not in mood for going out, I called a “Hallo waterpipe” provider and they immediately brought me a ready-to-use waterpipe. (Current smoker, 18 year old)

#### Mass media silence respecting WTS

Some participants referred to mass media silence as another factor contributing to WTS by young women. They noted that there are no well-organized WTS-related educational programs in the national media.

The mass media has adequately covered addictive drugs such as marijuana so that the public has completely been informed about its harmful effects. However, there are limited educational programmes on WTS in the television and they did not cover the whole reality of WTS in their movies. (Current smoker, 26 year old)

Participants noted that not only the mass media treats WTS with silence, but also shows movies and serials which promote WTS.

For instance, a popular serial depicted an actress who was smoking waterpipe in a family circle. Such depiction of WTS by a woman promotes WTS among people, particularly girls. (Current smoker, 22 year old)

On the other hand, some participants referred to anti-WTS campaigns in media in which well-known artists and athletes give anti-WTS messages to the public.

The “Turn waterpipes into flowerpots campaign” was very good. The administrators of this campaign employed artists and athletes and I think it was effective in sensitizing people to WTS. These programmes can broaden people’s knowledge about the harmful effects of WTS and thereby, can reduce WTS. (Mother, 42 year old)

### Common sociocultural traditions

Another main sociocultural factor contributing to WTS was common sociocultural traditions. This main category included two subcategories, namely WTS as a norm transgression and social acceptance of WTS by adolescent and young adult women.

#### WTS as a norm transgression

Our participants’ experiences showed that WTS by girls and women is considered as deviation from social norms in some areas.

WTS by girls is not approved by our society; yet, some girls go to hookah bars for this deviant behavior. (Female teacher, 50 year old)

A waterpipe smoking girl said,

Waterpipe smoking by girls is associated with social stigma and infamy. If my relatives see me while I’m smoking waterpipe, it would lead to bad troubles for me. For instance, it can cause me troubles in marriage in the future. However, here in a hookah bar, you are alone and are less likely to be seen by others. (Current smoker, 22 year old)

#### Social acceptance of WTS by women

In some communities in Iran, WTS is traditionally used in different parties and ceremonies. In fact, WTS is more acceptable than other tobacco-smoking methods in the Iranian society. Some participants noted that their WTS behaviour was formed by such traditions.

When I was a child, I went to a religious memorial ceremony where there was a row of waterpipe for old women. There, I was tempted into WTS and smoked waterpipe for the first time. (Current smoker, 14 year old)

Some participants recommended the use of healthy entertainment activities in ceremonies as a strategy for reducing the social acceptance of WTS by women.

In our night parties, we play traditional games. Brain games are also good substitute for WTS. Such games can reduce the acceptance of WTS in the society and families. (Mother, 39 year old)

### Governmental policies and regulations

Ineffective enforcement of WTS-related regulations and inadequate supervision of the production, import, and export of tobacco, along with inadequate control over WTS in hookah bars significantly contribute to the increasing prevalence of WTS among women. The two subcategories of this category were ineffective enforcement of WTS-related regulations and inadequate supervision of tobacco production, supply, import, and export.

#### Ineffective enforcement of WTS-related regulations

Some participants noted that the wide variety of WTS-related regulations and their ineffective enforcement have reduced WTS providers’ and users’ compliance to these regulations.

They enacted a regulation which forbade WTS. Then, another regulation was enacted to make WTS in hookah bars legal. After that, they re-forbade WTS. Such non-strict regulations cause both WTS providers and users not to take the regulations serious and thus continue WTS. (Smoking control staff, male, 33 year old)

According to the participants, WTS-related regulations are just enacted, and are not effectively enforced.

Regulations forbid the selling of tobacco to people under eighteen. However, such regulations are not effectively enforced. (Boyfriend, 23 year old)

Non-strict regulations and their ineffective enforcement have significantly increased the number of hookah bars in Iran and made WTS services more easily accessible.

Hookah bars are available everywhere. For instance, there is several traditional hookah bars near our university, where we easily access and smoke waterpipe. Such easy access to WTS services has promoted WTS among us, the girls. (Current smoker, 19 year old)

Poor tax policies on tobacco has also made WTS less expensive than the other types of recreational activities in Iran.

WTS is the most cost-effective recreational activity. There is no high tax on WTS and most people can afford it, while not everybody can afford the costs of going to amusement parks. (Current smoker, 25 year old)

#### Inadequate supervision of tobacco production, supply, import, and export

Some participants attributed the increasing prevalence of WTS in Iran to governmental support for tobacco use.

The government can easily eradicate WTS like the eradication of many other things; however, it unfortunately supports it because tobacco is a domestic product. (Current smoker, 22 year old)

Ineffective supervision of the enforcement of regulations related to tobacco production, supply, import, and export also significantly contributes to the prevalence of WTS among adolescent and young adult women.

We have fundamental problems. The easy and non-supervised import and export of tobacco increase WTS in our country. (Current smoker, 25 year old)

Moreover, regulations and supervisions of WTS by women are not effective and hence, stricter policies are needed to reduce WTS prevalence among women.

The cancellation of WTS in hookah bars and restaurants is impossible. The government needs to develop more effective policies. For instance, the creation of more employment opportunities for young people by the government can help reduce WTS prevalence. (Current smoker, 26 year old)

## Discussion

The current research is among the handful of qualitative studies which explored sociocultural factors contributing to WTS among Iranian adolescent and young adult women. Findings revealed that the main sociocultural factors contributing to WTS among Iranian adolescent and young adult women were waterpipe glamorization by its producers and sellers, media advertisement or silence, common sociocultural traditions, and governmental policies and regulations. Although the main part of the data were extracted from the interviews with main participants, i.e., young women, the results of data analysis during the study directed us towards interviewing other people including main participants’ family members, friends, WTS providers, and smoking control staff in order to collect more in-depth data. Findings indicated that participants’ main concerns were different from each other. For instance, the main concern of WTS providers was to attract more customers, while the main concern of smoking control staff was related to the lack of clear smoking control guidelines.

Findings indicated that waterpipe producers use different techniques to glamorize waterpipe and its accessories in order to tempt people into WTS. Similarly, a former study reported that the key success factor of tobacco industry is innovations in tobacco products and tobacco-using devices (Nakkash et al., 2011). Factors such as innovative designs for tobacco packing and flavouring are also effective in attracting young people and women to tobacco smoking (Afifi et al., 2013; Sharma et al., 2014). These findings denote that the tobacco industry use subtle and innovative techniques to attract adolescent and young adult women’s attention and tempt them into WTS.

Besides innovative techniques used by waterpipe producers, our findings indicated that WTS providers also use different techniques to market WTS, attract more customers, and tempt young women into WTS. In line with these findings, previous studies found that the owners of restaurants and coffee shops consider WTS as a profitable business and hence, use different techniques to compete with each other for attracting more customers (Afifi et al., 2013; Nakkash et al., 2011). Our participating adolescent and young adult women referred to the beautiful interior decoration and the pleasant environment of hookah bars as factors tempting them into WTS. The current findings revealed that WTS providers and waterpipe producers and sellers resorted to techniques such as waterpipe glamorization and using women’s favourite-coloured lighting and decoration in hookah bars in order to tempt them into WTS. Previous studies also reported the role of environmental decoration and lighting in attracting people to hookah bars and tempting them into WTS (Nakkash et al., 2011; Sharma et al., 2014).

Our findings also showed that hookah bars attempt to attract more customers through providing other services such as traditional foods, different drinks, confections, nuts, free high-speed internet, and live music. This is in line with the findings of two studies in the USA which showed that hookah bars provide their customers with services such as different music, foods (Kassem et al., 2015; Sharma et al., 2014), alcoholic drinks, coffee, tea, free internet services, dancing opportunities, and computer services for student customers to do their homework (Kassem et al., 2015). The “Hallo waterpipe” service was another factor contributing to WTS among young women in the present study. An earlier study also reported the same finding (Afifi et al., 2013). Special discounts on waterpipe, particularly for students, were another finding of the study. To the best of our knowledge, none of the previous studies in this area reported waterpipe discount for students.

Some of our participants attended hookah bars and smoked waterpipe in order to violate social norms. For instance, they chose hookah bars because these places provided them with private places for spending time with their intimate friends along with offering waterpipe. Evidence also confirms that going to traditional restaurants and coffee shops increases the likelihood of WTS (Kassem et al., 2015; Khalil et al., 2013). Some of our participants considered WTS by women as a norm transgression. Similarly, a former study reported that WTS by women is a shameful deviant behaviour which is against social norms and noted that women who smoke waterpipe are not dignified (Khalil et al., 2013).

Findings showed that media advertisement was another factor contributing to WTS among adolescent and young adult women. Some TV shows depict women who smoke waterpipe and thereby, promote WTS. Moreover, non-official media, such as Instagram and Telegram, displays advertisements for WTS. A study reported that the image of waterpipe advertised by media was attractive, sexy, and soothing (Nakkash et al., 2011). Another study showed that the Instagram and other social networks promote WTS through depicting positive images and relating it to glamorous lifestyle, disco attendance, alcohol consumption, and sexual allure (Ben Taleb et al., 2019). Moreover, compared with videos on cigarette smoking, WTS-related videos are more commonly watched, liked, and commented in YouTube (Carroll et al., 2013). In social networks, waterpipe smokers share posts about their WTS and are exposed to WTS posts shared by their friends (Link et al., 2015). Tobacco and waterpipe companies also resort to non-official media for advertising their products. At the same time, official media silence about WTS fuels misconceptions about the fewer harmful effects of WTS compared with cigarette smoking (Gathuru et al., 2015; Ssewanyana et al., 2018), and thereby, promotes its use by different people, including women.

Although some of our participants considered WTS by women as a violation of social norms, some other participants noted that the traditional use of waterpipe in some areas promotes WTS by women. Our participants also referred to WTS in some family circles and parties. WTS is also common in both wedding and burial ceremonies in some other countries, where lots of young people are present (Ssewanyana et al., 2018). Many Iranians view WTS as a recreational activity and do not have negative attitudes towards it. Similarly, a study in Syria reported that although cigarette smoking is a social taboo, WTS is considered as a socially acceptable traditional experience (Hammal et al., 2008).

Study findings also indicated ineffective enforcement of WTS-related regulations and inadequate supervision of tobacco production, supply, import, and export as factors contributing to WTS among adolescent and young adult women. In line with these findings, a study reported that despite protective policies and regulations for limiting adolescents’ access to tobacco, these policies and regulations were not effectively enforced because tobacco sellers had close relationships with some governmental authorities and were able to conduct their illegal business (Ssewanyana et al., 2018). Another study reported that unclear, unstable, and non-strict WTS-related regulations in different countries have facilitated people’s access to WTS and have given them misconceptions about WTS safety (Martinasek et al., 2011). The World Health Organization introduced the Framework Convention on Tobacco Control (FCTC) in order to control tobacco use throughout the world. Although many countries, including Iran, accepted to adhere to this convention, not all these countries show close adherence to it. Moreover, tobacco control policies in the Eastern Mediterranean region mostly focus on cigarette smoking control (Salloum et al., 2017).

Most participants referred to the low price of WTS as a significant factor contributing to their WTS. One of the reasons for the inexpensiveness of WTS in Iran is low tax on tobacco products. Comparison of the Eastern Mediterranean countries respecting tobacco control programmes revealed that Iran obtained the highest score for the MPOWER six policies on tobacco control; nonetheless, Iran has not completely been successful in enforcing the smokeless environment law and imposing tax on tobacco products (Heydari et al., 2017). In line with our findings, a study reported that the low price and the easy accessibility of WTS significantly contribute to its high prevalence (Afifi et al., 2013). However, a study on students at a university in the USA reported the expensiveness of WTS as a barrier to its use (Kassem et al., 2015). This finding confirms our findings regarding the significant contribution of WTS low price to its wide use in Iran.

## Limitations and strengths

Some hookah bar owners were reluctant to participate in the study because they were concerned with the negative effects of their participation on their business. Moreover, participating women might have shared mostly their socially desirable experiences and avoided to share some aspects of their real-world experiences due to the sensitivity of the study subject matter. In addition, this study may have limitations in generalizability of findings because the sample size was relatively small. On the other hand, one of the strengths of the study was the wide diversity of the study participants respecting their ethnicities, educational levels, and sociocultural status. This wide diversity improved the generalizability of the study findings.

## Conclusion and implications

The findings of the present study provide a new series of sociocultural factors contributing to WTS among adolescents and young adult women in Iran. These factors include waterpipe glamorization by its producers, different customer attraction techniques used by WTS providers, common WTS-related sociocultural traditions, people’s misconceptions and lack of knowledge about WTS due to media advertisement and silence, and governmental policies and regulations. A novel and interesting finding of this study is related to the techniques used by WTS providers and waterpipe producers and sellers for attracting more female customers and tempting them into WTS. Examples of these techniques were waterpipe glamorization and using women’s favourite-coloured lighting and decorations in hookah bars. The diversity of WTS-related factors highlights the importance of using interdisciplinary context-based multidimensional strategies for its prevention. These strategies may include public education through media, strict supervision of tobacco and waterpipe import, export, and selling, ample employment opportunities for young people, careful planning for adolescents and young adult people’s leisure time, high tax on tobacco, and strict regulations for banning tobacco provision to adolescents, preventing WTS in public places, and preventing tobacco advertisement.

The present study provides useful information for governors, healthcare authorities, and healthcare policymakers to be used in developing strategies to minimize and prevent WTS among women. Moreover, it provides a deep insight to develop new hypotheses concerning the effects of smoking control regulations. Accordingly, interventional studies are mandated to evaluate the effects of different legal interventions on WTS among women. Moreover, studies are needed to produce reliable statistical data on the prevalence of WTS, and also to determine the personal and behavioural motives for WTS and its cessation.
